# Reactivity Profiling for High-Yielding Ynamine-Tagged Oligonucleotide Click Chemistry Bioconjugations

**DOI:** 10.1021/acs.bioconjchem.4c00353

**Published:** 2024-10-10

**Authors:** Frederik Peschke, Andrea Taladriz-Sender, Allan J.B. Watson, Glenn A. Burley

**Affiliations:** † Department of Pure and Applied Chemistry, University of Strathclyde, Thomas Graham Building, 295 Cathedral Street, Glasgow G1 1XL, U.K.; ‡ Strathclyde Centre for Molecular Bioscience, University of Strathclyde, Glasgow G1 1XL, U.K.; § EaStCHEM, School of Chemistry, University of Saint Andrews, North Haugh, Fife, St Andrews KY16 9ST, United Kingdom

## Abstract

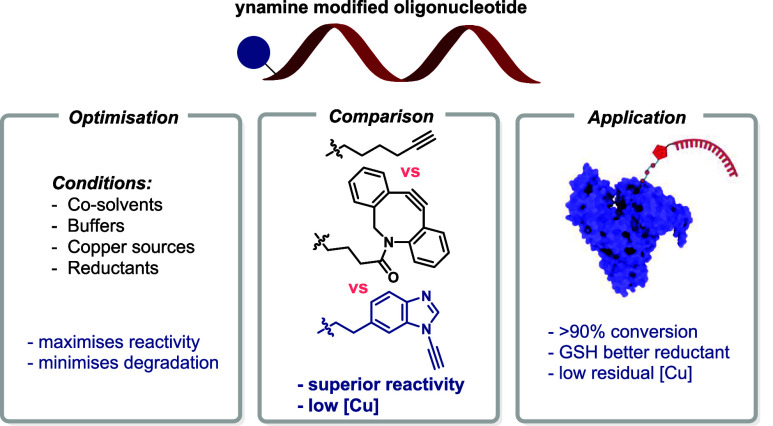

The Cu-catalyzed azide–alkyne cycloaddition (CuAAC) reaction is a key ligation tool used to prepare bioconjugates. Despite the widespread utility of CuAAC to produce discrete 1,4-triazole products, the requirement of a Cu catalyst can result in oxidative damage to these products. Ynamines are superior reactive groups in CuAAC reactions and require lower Cu loadings to produce 1,4-triazole products. This study discloses a strategy to identify optimal reaction conditions for the formation of oligodeoxyribonucleotide (ODN) bioconjugates. First, the surveying of reaction conditions identified that the ratio of Cu to the choice of reductant (i.e., either sodium ascorbate or glutathione) influences the reaction kinetics and the rate of degradation of bioconjugate products. Second, optimized conditions were used to prepare a variety of ODN-tagged products and ODN-protein conjugates and compared to conventional CuAAC and Cu-free azide–alkyne (3 + 2)cycloadditions (SPAAC), with ynamine-based examples being faster in all cases. The reaction optimization platform established in this study provides the basis for its wider utility to prepare CuAAC-based bioconjugates with lower Cu loadings while maintaining fast reaction kinetics.

## Introduction

The Cu-catalyzed alkyne–azide (3 + 2)cycloaddition (CuAAC) or “click” reaction is a ligation and labeling approach used extensively throughout medicinal chemistry and chemical biology.^1−4^ The combination of the small size of alkyne and azide reactive groups, their ease of incorporation into biomolecules, and the exclusive formation of a 1,4-triazole product render the CuAAC reaction one of the vanguard bio-orthogonal ligation tools used to prepare protein, nucleic acid, and glycoside-based bioconjugates.^5−7^ The utility of the CuAAC reaction in the postsynthetic modification of nucleic acids is of particular value as it provides a facile means to label oligodeoxyribonucleotides (ODNs) with reporter groups (e.g., fluorophores, spin labels, fluorinated reporters, and affinity tags)^8−12^ and for the formation of bioconjugates.^13,14^ This is underpinned by the ease of preparing alkyne-based phosphoramidites and triphosphates for their incorporation into DNA or RNA, either by solid phases or enzymatic syntheses.^15−18^

While Cu-free bio-orthogonal approaches for DNA/RNA labeling such as the strain-promoted alkyne–azide (3 + 2)cycloaddition (SPAAC)^19^ or inverse electron demand Diels–Alder (IEDDA) proceed with faster reaction kinetics, these approaches are often limited to postsynthetic modification strategies as their corresponding phosphoramidite building blocks are not stable under traditional oligonucleotide solid-phase conditions^20^ and can react with thiol groups, such as cysteine residues.^21−23^ Finally, SPAAC and IEDDA approaches can form inseparable regioisomeric mixtures, which could be problematic for downstream medical applications of bioconjugates, as each regioisomer could possess distinct pharmacological profiles. Taken collectively, the CuAAC reaction remains one of the methods of choice for oligonucleotide conjugation over Cu-free alternatives.

**Figure 1:**
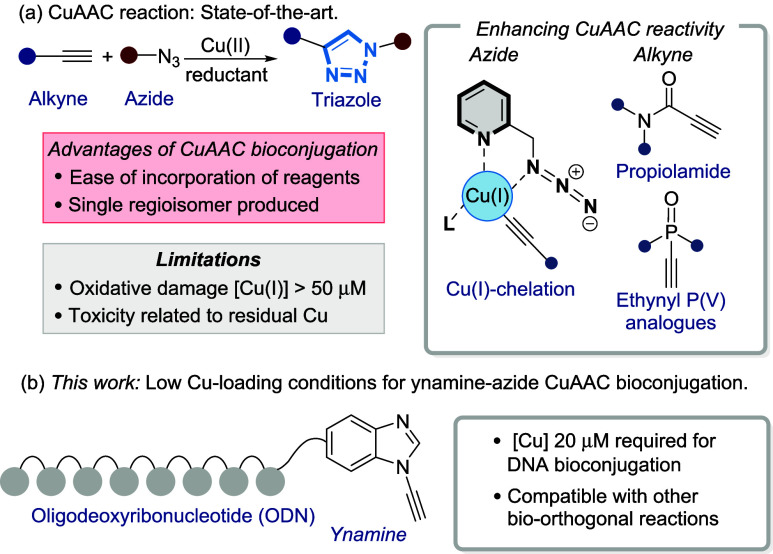
(a) The current state-of-the-art of the CuAAC reaction. (b) The ynamine-azide (3 + 2)cycloaddition as an approach for DNA bioconjugation using low Cu loadings.

Despite the extensive use of the CuAAC reaction for the modification of nucleic acids,^24,25^ one major limitation is the need to use excess Cu catalyst with respect to the alkyne and azide reagents to afford ligation products in high yield.^26^ This is deleterious as excess Cu results in the onset of oxidative damage (e.g., 8-oxo-G), particularly to guanine-rich sequences (Figure 1a).^5,27^ The onset of oxidative damage can lead to strand breaks, and while it is often mentioned in the literature,^28-30^ it is rarely quantified^31^ as a function of the CuAAC reaction conditions.^27^ Strategies to minimize oxidative damage to nucleic acids have ranged from the use of Cu ligands and Cu nanoparticles through to the use of Cu-chelating picolyl azides as well as degassing of the reaction medium.^32−35^

While these strategies can result in a reduction of Cu loadings, an alternative approach is to understand why excess Cu loadings are needed by studying the mechanism of the CuAAC reaction. The proposed rate-determining step (RDS) of the conventional CuAAC reaction is the formation of a binuclear Cu-acetylide species, which suggests that using activated alkynes might be beneficial.^36−38^ Previous work by the Finn group identified propiolamides as superior alkyne substrates for CuAAC-mediated ligations performed in aqueous buffers.^39^ However, the susceptibility of propiolamides to undergo Michael addition with thiols can be a limitation for their wider applicability as a reagent for CuAAC-mediated ligations.^40^ As a result, efforts toward lowering Cu loadings for CuAAC-based ligations will require developing more reactive alkyne groups that are stable under physiologically relevant conditions, display sufficient stability against nucleophiles (e.g., thiols), and are also compatible with their incorporation into biomolecules.

Placement of heteroatoms in direct conjugation with the sp C≡C bond is one strategy to enhance alkyne reactivity.^41,42^ Of the heteroatom alkynes explored in CuAAC ligations,^43−45^ heteroarylalkynes (ynamines) have shown superior reactivity in CuAAC reactions.^46−49^ Their enhanced reactivity has been demonstrated in the chemoselective formation of peptide and ODN ligation products in the presence of terminal alkynes and cyclooctynes either in batch or in flow (Figure 1b).^49-51^

Herein, we undertake a systematic analysis of the reaction conditions of the Cu-catalyzed ynamine-azide (3 + 2)cycloaddition reaction when used for ODN ligation and interrogate the potential oxidative damage of the bioconjugate product. Comparative analyses of the ynamine-CuAAC ligation with traditional CuAAC and SPAAC-based ligations show that the ynamine-CuAAC reaction achieves superior results in ODN labeling to form discrete bioconjugates. We show that the choice of organic cosolvent, buffer, and Cu ligand influences ynamine reactivity as well as the stability of the ODN toward oxidation. Finally, we outline an optimized workflow which maximizes ligation yields of ODNs with small molecules and bovine serum albumin (BSA) while minimizing oxidative degradation, dispelling the need to degas the reaction.

## Results and Discussion

### Experimental Approach

**Figure 2:**
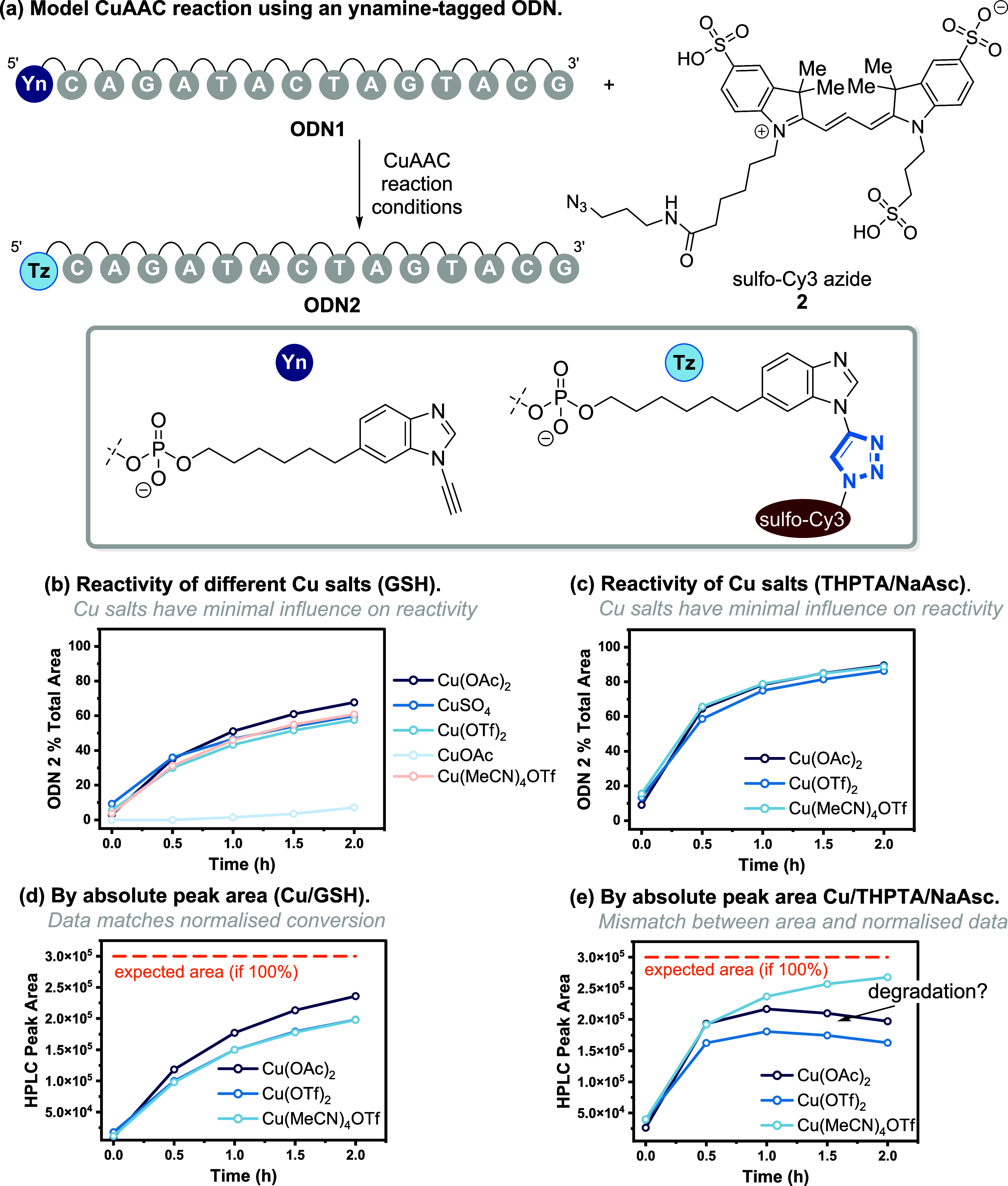
(a) General reaction scheme for the conversion of **ODN1** with **2** using either Cu/GSH or Cu/THPTA/NaAsc. (b) The % total area of **ODN2** after 2 h as a function of cosolvent using Cu/GSH. (c) The % total area of **ODN2** after 2 h as a function of buffer using Cu/THPTA/NaAsc. (d) Selected data from (b) plotted as an HPLC peak area. (e) Data from (c) plotted by the HPLC peak area. Conditions for Cu/GSH: **ODN1** (10 μM), sulfo-Cy3-azide **2** (20 μM, Cu(OAc)_2_ (50 μM), and GSH (50 μM) in H_2_O (20 mM MgCl_2_). Conditions for Cu/THPTA/NaAsc: **ODN1** (10 μM), sulfo-Cy3-azide **2** (20 μM), Cu(OAc)_2_ (10 μM), THPTA (50 μM), and NaAsc (1 mM) in H_2_O (20 mM MgCl_2_); 10% MeOH as cosolvent for Cu(MeCN)_4_OTf due to poor solubility.

The overall objective of this study was to assess how the yield of Cu-catalyzed ynamine-azide (3 + 2)cycloadditions is influenced by the reaction conditions for the click labeling of ODNs. Although our previous work identified Cu(OAc)_2_ and GSH as an efficient catalyst/ligand system compared to the conventional CuSO_4_/THPTA/NaAsc system,^51,52^ a more detailed understanding of how the choice of buffer, reductant, Cu ligand, and cosolvent all play a role in ODN modification was lacking. Our optimization workflow used a representative ynamine-modified ODN (**ODN1**) and a fluorescent Cy3 azide **2** as the corresponding reagent pair (Figure 2a). The % conversion of the CuAAC reaction to **ODN2** was determined by IPRP-HPLC, initially by dividing the product peak area by the total peak area to capture any potential degradation products or side reactions. The synthesis of **ODN1** was achieved by coupling a ynamine group to the 5′ end by automated solid-phase synthesis using phosphoramidite **S2**.

### Optimization of Ynamine-Azide ODN CuAAC Bioconjugation

The influence of organic cosolvent (5–10%) in phosphate buffer (1× DPBS) was first explored. This involved surveying water-miscible organic solvents (e.g., MeOH, MeCN, and DMSO) as well as fluorinated solvents (Figure S7a). Fluorinated solvents were added to the cosolvent screen as they offer a unique balance of hydrophobicity and polarity,^53^ which we surmised could be used to fine-tune reactivity of CuAAC bioconjugation reactions.^54^ Overall, the addition of cosolvent decreased conversion to **ODN2** when compared to undertaking the reaction in 100% 1× DPBS buffer (∼50% after 2 h, Figure S7a). When decreasing the percentage of cosolvent from 10% to 5%, conversion to the **ODN2** product increased, especially when HFIP was used (i.e., an increase from 17% to 41% after 2 h). As a result of this screen, we conclude that the addition of an organic cosolvent should be minimized, particularly if a water-soluble azide is used to react with an ODN under CuAAC conditions.

The influence of the buffer type on the conversion of **ODN1** into **ODN2** under CuAAC reaction conditions was then explored (Figure S7b). A 1× DPBS phosphate buffer was first compared to using water as well as the influence of different concentrations of MgCl_2_.^26^ Using **ODN1** as the corresponding reagent, [MgCl_2_] (0.5, 10, and 20 mM) in 1× DPBS buffer did not greatly influence the conversion to **ODN2** (Figure S7b). However, when no buffer was used, the addition of MgCl_2_ increased the conversion from 10% to ∼70% after 2 h. As a result of this screen, an aqueous solution containing 20 mM MgCl_2_ was used for the following experiments.

Next, the influence of the Cu source, Cu ligand, and reductant was explored. In almost all examples, similar levels of conversion to **ODN2** were observed using the Cu/GSH system (Figure 2b). One exception to this trend was CuOAc, which was likely due to the poor aqueous solubility of this salt. When the Cu/THPTA/NaAsc system was used (Figure 2c), conversion to **ODN2** increased when compared to the Cu/GSH system even though 5-fold less [Cu(OAc)_2_] was used. However, the amount of **ODN2** present was significantly lower than expected (Figure 2e), something that was not observed to the same extent with the Cu/GSH system (Figure 2d). Insidiously, this drop in the raw HPLC area was not observed when calculating conversions by the total area and was noticed only when inspecting the raw HPLC data. We speculated that the decrease in the peak area might be due to Cu-mediated degradation, which was less pronounced when Cu(MeCN)_4_OTf was used compared to Cu(OAc)_2_ or Cu(OTf)_2_—possibly due to the addition of 10% MeOH to ensure the solubility of the Cu(I) salt.^31,55^ As a consequence, we changed our procedure to calculating conversion by dividing the product area (**ODN2**) by the starting **ODN1** area at *t* = 0 min, an approach which reflected conversion and degradation much more accurately.

**Figure 3:**
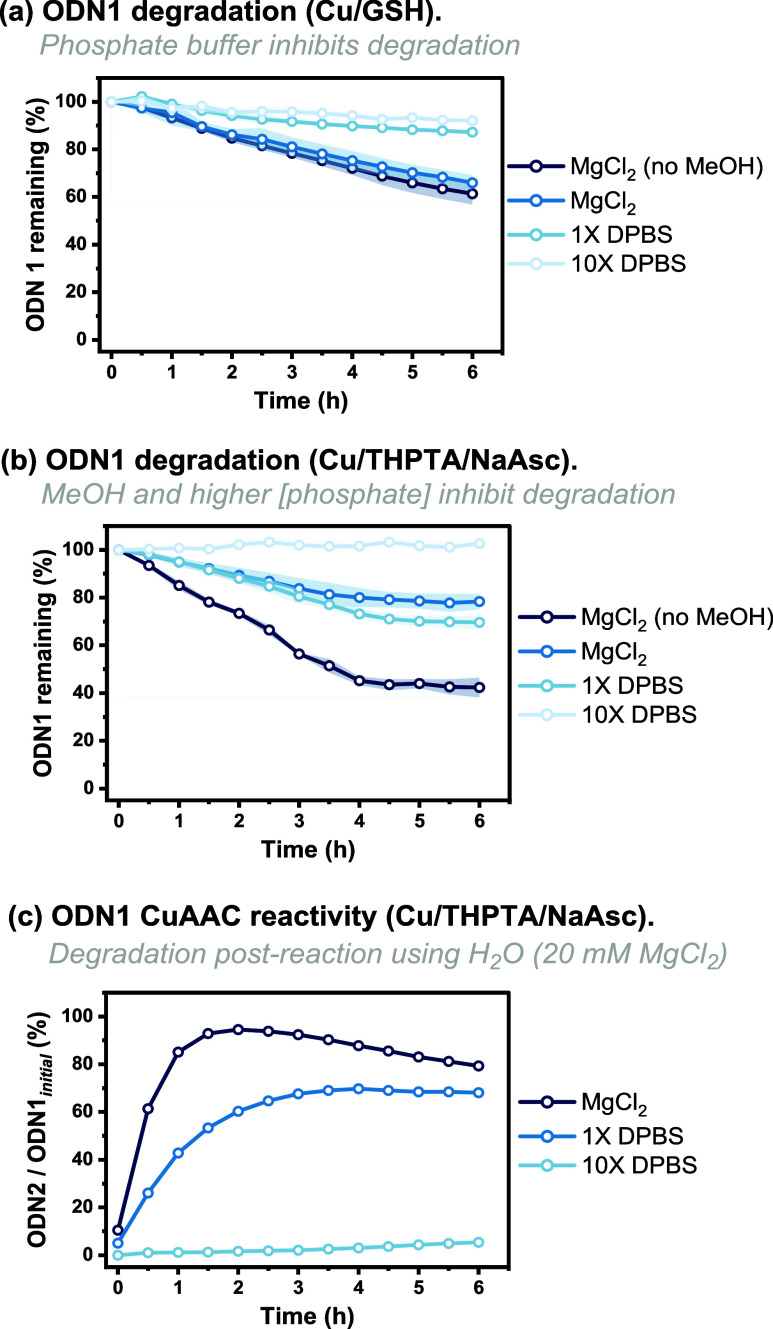
Degradation profile of **ODN 1** in the presence of (a) Cu/GSH and (b) Cu/THPTA/NaAsc systems using different buffers and cosolvents. Reaction conditions (Cu/GSH): **ODN1** (10 μM), Cu(OAc)_2_ (50 μM), and GSH (50 μM) in buffer containing 10% MeOH unless otherwise indicated; (Cu/THPTA/NaAsc): **ODN1** (10 μM), Cu(OAc)_2_ (10 μM), THPTA (50 μM), and NaAsc (1 mM) in 10% MeOH unless otherwise indicated. Reactions in (c) contain 20 μM (**2**).

These findings prompted us to explore the interplay between the loss of **ODN1** over time under the reaction conditions versus the rate of formation of **ODN2** using either Cu/GSH or Cu/THPTA/NaAsc to catalyze the reaction. Several key trends were observed. First, the degradation profile of **ODN1** induced by the Cu/GSH (Figure 3a) was less than that of Cu/THPTA/NaAsc (Figure 3b) even though a 5-fold increase in Cu was used for the Cu/GSH system. Second, the addition of 10% MeOH increased the stability of **ODN1** (i.e., from ∼50% to ∼80% using H_2_O and 20 mM MgCl_2_) when NaAsc was used as the reductant (Figure 3b). This effect was less pronounced when Cu/GSH was used (Figure 3a). Third, using 1× DPBS reduced degradation in a concentration-dependent manner, with this effect being more pronounced when Cu/THPTA/NaAsc was used. **ODN1** is then stable over 6 h when 10× DPBS was used. Finally, the degradation profiles of **ODN1** differed. In the Cu/THPTA/NaAsc system, the rate of degradation correlated with NaAsc consumption with degradation stopping once NaAsc was fully consumed (*t* = 4 h). In contrast, the GSH system led to steady degradation of **ODN1** over time, which was less influenced by buffer and cosolvent parameters. Of note, degradation is not a function of the ynamine moiety, as similar reaction profiles were also observed for alkyne containing oligo **ODN3** (Figure S8).

Since the degradation of **ODN1** was minimal using Cu/THPTA/NaAsc in 10× DPBS, further studies were undertaken using 10% MeOH as a cosolvent under CuAAC conditions with azide **2** to form **ODN2** (Figure 3c). Conversion to **ODN2** was low when 10× DPBS was used, reaching ∼10% conversion after 6 h. Using 1× DBPS resulted in ∼70% conversion to **ODN2**, whereas 20 mM MgCl_2_ in water mixture resulted in conversion to **ODN2** ∼ 90% after 2 h. However, the peak area of **ODN2** using HPLC steadily declined after 2 h, which we assume was due to degradation after the completion of the reaction, likely due to consumption of **ODN2**.

This degradation was likely caused by cycles of reduction and oxidation mediated by NaAsc and oxygen, possibly producing reactive oxygen species in the reaction mixture.^56^ Taken collectively, these experiments show that (i) the reactivity of ynamine and azides reagents under CuAAC ligation conditions is influenced by the buffer used and (ii) there is a trade-off between faster reaction kinetics reconciled with the onset of ODN degradation products.

Since NaAsc was key to ensuring rapid reactivity while minimizing degradation, we utilized the design of experiments (DoE) to gain a more detailed understanding of how [NaAsc] as well as [THPTA] influences reactivity. A full factorial DoE series was used with one center point (Table S4). [Cu(OAc)_2_] was fixed at 10 μM, with [THPTA] varied between 10 and 50 μM and [NaAsc] between 100 and 1000 μM. Two main trends were observed. First, a [NaAsc] of 100 μM was sufficient to reach the maximum conversion (∼80%), and the highest concentration of NaAsc tested (1 mM) consistently led to slightly lower conversions (Figure S9). Second, higher [THPTA] led to a slightly faster reaction rate.

**Figure 4:**
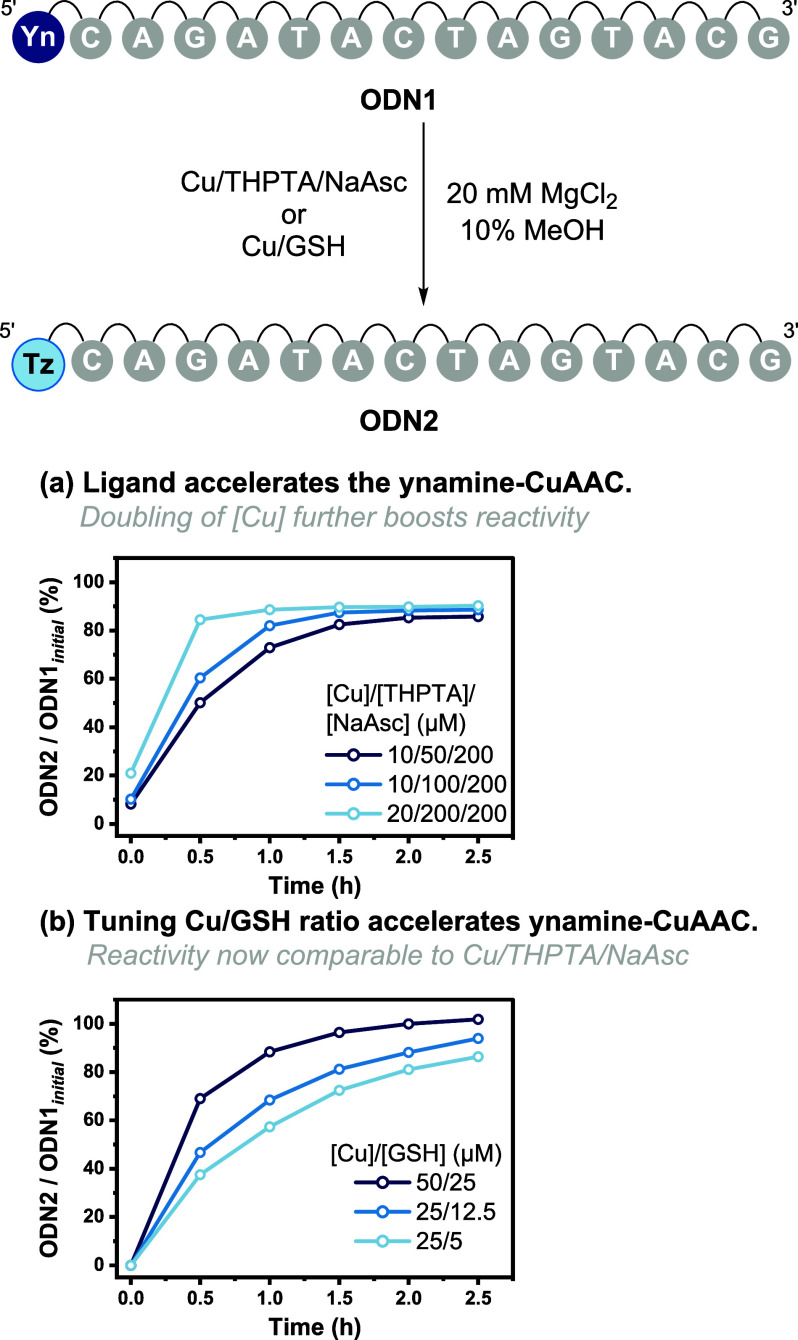
Optimization of the CuAAC reaction using **ODN1** and **2** to form **ODN2** using either Cu/THPTA/NaAsc or Cu/GSH. (a) Increasing [THPTA] and [Cu] increases the reaction rate. Conditions: **ODN1** (10 μM), azide **2** (20 μM), THPTA (50 or 100 μM), Cu(OAc)_2_ (10 or 20 μM), and NaAsc (200 μM) in H_2_O (20 mM MgCl_2_, 10% MeOH). (b) Changing Cu:GSH to 2:1 increases reactivity. Conditions: **ODN1** (10 μM), azide **2** (20 μM), GSH (5, 12.5, or 25 μM), and Cu(OAc)_2_ (25 or 50 μM) in H_2_O (20 mM MgCl_2_, 10% MeOH).

Further fine-tuning of the reaction conditions identified that increasing the Cu-stabilizing ligand THPTA to 10 equiv while simultaneously increasing [NaAsc] to 200 μM further increased the reaction rate (Figure 4a). Finally, doubling of the [Cu] from 1 to 2 equiv led to >80% conversion of **ODN1** to **ODN2** after 30 min. We then optimized the CuAAC reaction by using Cu(OAc)_2_ and GSH (Figure 4b).

Based on our previous results,^51^ we reasoned that lowering the [GSH] while keeping [Cu] constant (i.e., increasing the Cu:GSH ratio) would result in a faster reaction rate. Indeed, this was observed, and [Cu] was halved from 50 to 25 μM while simultaneously increasing the reaction rate (when compared to Figure 2b) by decreasing [GSH] to 5 μM (a ratio of Cu:GSH of 5:1). An increase in Cu:GSH to 2:1 further increased conversions. Since minimal degradation was observed, [Cu(OAc)_2_] was increased to the original value of 50 μM, resulting in a conversion of >95% in 1.5 h.

### Reaction Scope of Cu-Catalyzed Ynamine-Azide (3 + 2)Cycloadditions to Form ODN Conjugates

**Figure 5:**
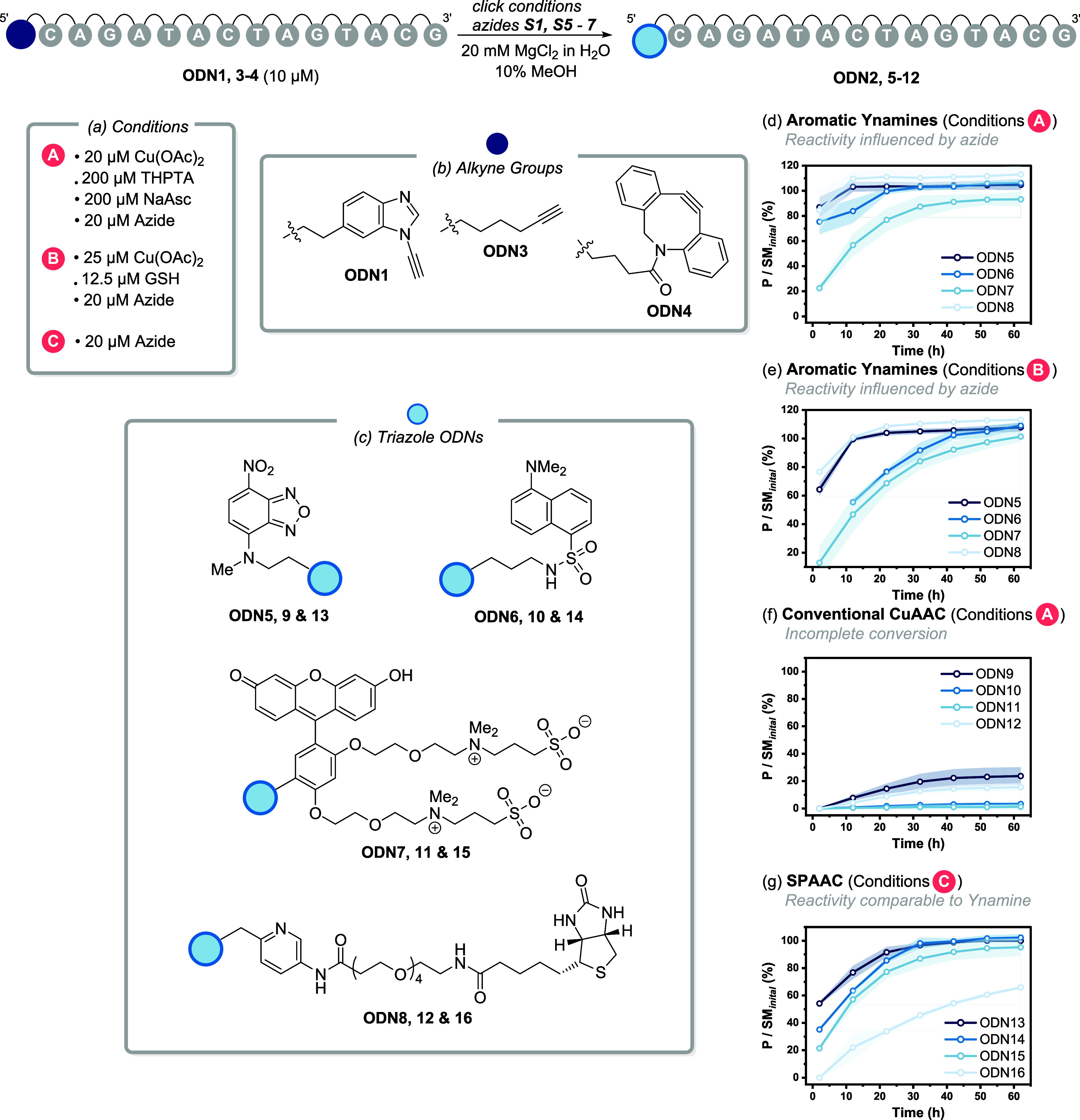
Reactivity comparison between ynamine-CuAAC, conventional CuAAC, and DBCO (a) Reaction conditions for all experiments. (b) Schematic of 5′ alkyne-modified oligonucleotides. (c) Schematic of formed triazole products. (d–g) Time courses of the formation of triazoles **ODN5**–**16** using the optimized reaction conditions. Shaded bands represent the standard deviation of triplicate experiments.

With the two sets of optimized ODN labeling conditions established, we explored the reaction scope across a panel of azides (see Figure S3 for structures **S1**, **S5**–**7**) with ynamine **ODN1**, traditional alkyne-modified **ODN3**, and DBCO **ODN4** (Figure 5; for full structures, see Figure S2 and Table S2). For the Cu/GSH system, we chose to use 25 μM Cu(OAc)_2_ despite being slightly slower than the optimal conditions in Figure 4b to balance the copper loading while still achieving adequate reactivity.

CuAAC ligation of **ODN1** with azides **S1**, **S5**, and **S7** to form triazole products (**ODN5**, **6**, and **8**) using Cu/THPTA/NaAsc reached >95% conversion within 10–20 min. Using Calfluor azide **S6** resulted in ∼90% conversion to **ODN7** within 1 h (Figure 5d). In comparison, utilizing the optimized Cu/GSH conditions led to a slower conversion of **ODN1** to form **ODN5**–**8**. However, all reactions were complete within 1 h (Figure 5e). In contrast, alkyne-modified **ODN3** reached only ∼15–20% conversion to **ODN9** and **ODN12** within 1 h (Figure 5f) when chelating azides were used. These conditions were optimized for the ynamine and employ a large excess of THPTA (10 equiv) which has been shown to inhibit the conventional CuAAC reaction.^57^ We therefore repeated the reaction of **ODN3** with picolyl azide **S7** using only 5 equiv of THPTA (Figure S10a) which is generally regarded as the optimum amount of ligand. The conversion improved from ∼15 to 22% and could only be brought to parity with the ynamine once the concentration of Cu(OAc)_2_ was increased 10-fold (Figure S10b). The Cu-free SPAAC reaction utilizing DBCO-modified **ODN4** was comparable to ynamine-modified **ODN1** (using Cu/GSH conditions) reaching >95% conversion within 1 h with the only exception being the biotin-picolyl azide-based conjugate **ODN16** (Figure 5g).

Several key trends can be observed from these data. First, the aromatic ynamine is more reactive than the conventional alkyne, regardless of the azides used. However, employing a chelating azide has an additional synergistic effect on the reactivity. Second, the conventional alkyne also benefits from the use of chelating azides. Third, the Cu-free reaction is a superior alternative to the conventional CuAAC reaction, and reactivity is influenced less by the azide. Based on these results, we would recommend either an ynamine or a DBCO-based click reaction for the modifications of oligonucleotides, especially if a conventional CuAAC must be used, and pairing the alkyne with a chelating azide reaction partner.

### Establishing a Robust Methodology for the Preparation of ODN-Protein Bioconjugates

Using our optimized reaction conditions for Cu-catalyzed ynamine-azide (3 + 2)cycloadditions using small molecule azides, we sought to expand the scope to prepare protein-ODNs. Protein-oligonucleotide conjugates have the potential for cell selective delivery of oligonucleotide payloads;^58−61^ thus, the development of robust and reproducible methods is vital for downstream application as next-generation biopharmaceuticals.^62^ BSA was used as a model protein for the optimization of this ligation reaction as it has a single surface accessible cysteine residue (Cys34) to install an azide group, and conjugation of oligonucleotides to BSA specifically has been shown to increase serum stability and cell uptake of oligonucleotide payloads.^63,64^

In contrast to the reaction optimization of the Cu-catalyzed ynamine-azide (3 + 2)cycloadditions using small molecule azides, proteins have numerous Cu chelating sites, resulting in low to moderate yields, and requiring an increase in Cu loadings and excess Cu-stabilizing ligands.^57,65−67^ The high Cu loading required to achieve reasonable yields of the protein-ODN conjugate can invariably lead to an increase in the formation of oxidative damage.^35,68^

**Figure 6:**
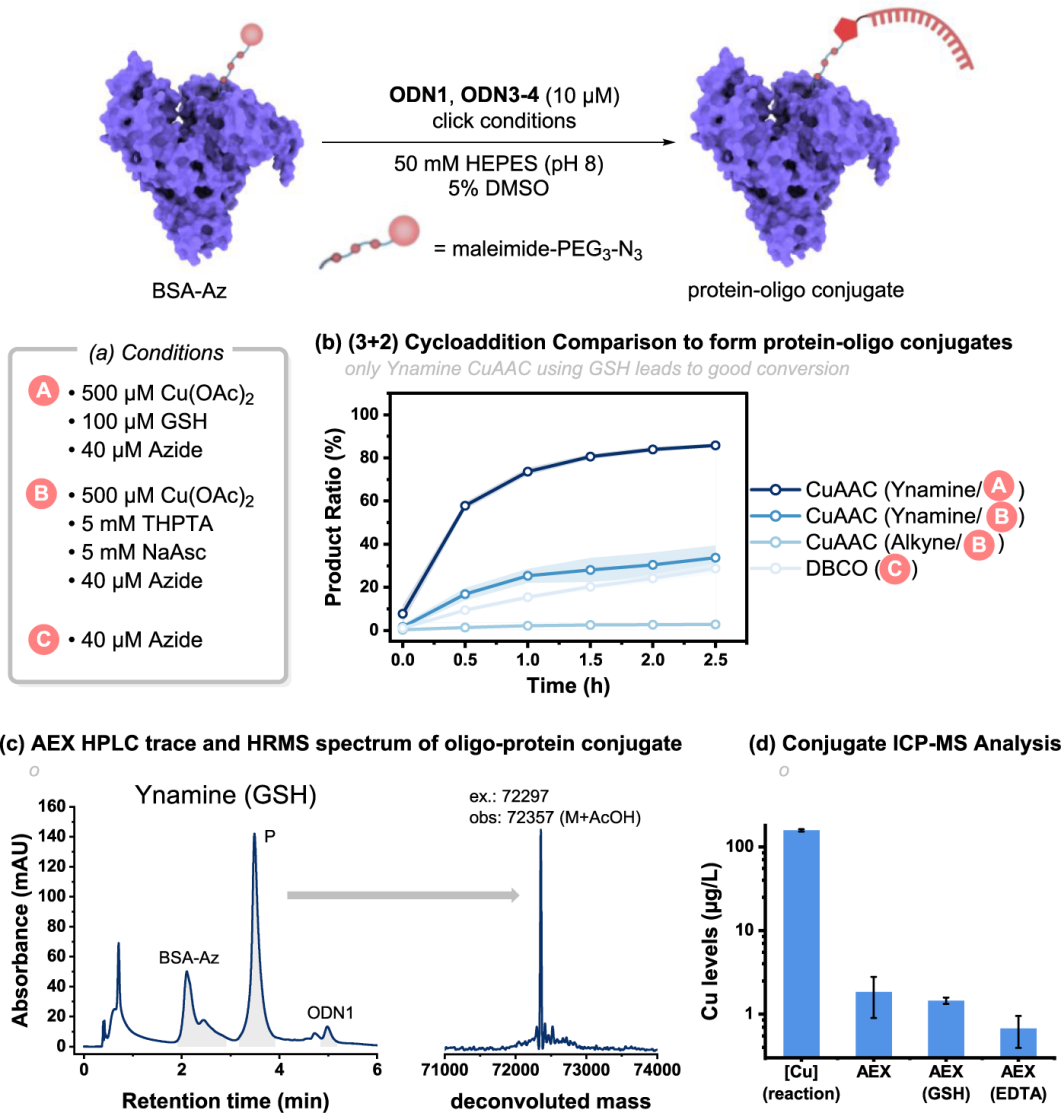
Comparison of several bio-orthogonal (3 + 2)cycloadditions for the formation of oligo-protein conjugate between BSA-Az and **ODN1**, **3**, and **4**. (a) Reaction conditions. (b) Time course for the reaction of **ODN1**, **3**, and **4** with BSA-Az utilizing the optimized reaction conditions. Shaded bands represent the standard deviation of triplicate experiments. (c) AEX chromatogram of ynamine-CuAAC at *t* = 2 h and deconvoluted HRMS of the conjugated product isolated by AEX. (d) ICP-MS analysis of the purified conjugate. AEX (GSH) = GSH addition (10 mM) prior to AEX purification; AEX (EDTA) = EDTA addition (10 mM) prior to AEX purification.

We surveyed reaction conditions to prepare a protein-ODN conjugate using a BSA-azide (**BSA-N**_**3**_) with a series of ODNs incorporating a 5′-ynamine (i.e., **ODN1**), 5′-alkyne (**ODN3**), and a corresponding cyclooctyne (**ODN4**) group (Figure 6a). BSA azide was prepared via conjugation of BSA with commercially available maleimide-PEG3-azide. Anion exchange HPLC (AEX-HPLC) or size exclusion chromatography (SEC) was used to monitor the conjugation reaction. The % conversion was calculated by dividing the peak area of the conjugate by the sum of **ODN1** and the conjugate peak area.

Poor reactivity was observed when lower [Cu] was used (Figure S11b). The highest conversions to the BSA-ODN conjugate were achieved by using 500 μM Cu(OAc)_2_. Another critical parameter was the azide concentration, with optimum conversions achieved when 4 equiv of **BSA-N**_3_ were used. DMSO as a cosolvent and 50 mM HEPES buffer were chosen to ensure protein solubility and were equivalent in terms of reactivity compared to the previous employed H_2_O/MgCl_2_ system (Figure S11a).

With the conditions for the ynamine-oligo protein conjugation optimized, we then surveyed how the efficiency of the ynamine-CuAAC reaction was influenced by the reductant (i.e., GSH and NaAsc) and the type of alkyne (i.e., a terminal aliphatic alkyne using CuAAC and NaAsc and the copper-free SPAAC reaction using DBCO). The Cu/GSH system achieved superior conversions to the BSA-ODN conjugate (i.e., >80% within 2.5 h), whereas both the NaAsc system and DBCO only reached ∼30% within the same time (Figure 6b).

The conventional alkyne CuAAC was poorly reactive under these conditions. After ∼18 h, the SPAAC reaction reached ∼70% conversion to the conjugated product. We confirmed the identity of the AEX-purified conjugation product by SEC-HRMS and investigated the amount of residual copper in the ynamine conjugation product by ICP-MS. A 100-fold reduction in copper content (compared to the starting reaction mixture) was observed after AEX purification (∼2 μg/L), which was lowered even further by incubation of the reaction mixture with EDTA prior to AEX purification (∼0.7 μg/L). Incubation with additional GSH as a Cu scavenger was less successful than EDTA addition and only reduced copper levels marginally (Figure 6d). Even though copper seems to be removed efficiently from the conjugate by AEX purification and EDTA addition, the amount of Cu required for the formation of the BSA-oligo conjugate is still high. From our results shown in Figure 5, we speculate that the use of a chelating azide could reduce the required copper loading further.

## Conclusions

In summary, we have shown that the use of 5′-tagged ynamine ODNs is a superior reactive group for bioconjugation via the Cu-catalyzed ynamine-azide (3 + 2)cycloaddition. Critical to the utility and high conversions to the triazole products when 5′-tagged ynamine ODNs are used is the need to survey a combination of solvent, Cu source, and reductant. DoE offers a streamlined method to optimize these bioconjugations and can be used to rapidly identify reaction conditions for the formation of a range of ODN bioconjugates, from small molecules through to proteins. These findings will expand the repertoire of the bioconjugation toolbox, particularly where single discrete products are required.

## Supporting Information Available

Full compound structures, experimental methods, compound characterization, HPLC assay protocol, oligo-protein conjugate mass spectrometry data, and additional figures (PDF)
