# Supplementary material for: Reactivity Profiling for High-Yielding Ynamine-Tagged Oligonucleotide Click Chemistry Bioconjugations
Source: Bioconjug Chem. 2024 Oct 10;35(11):1788–96. doi: 10.1021/acs.bioconjchem.4c00353 (PMC11583209; doi:10.1021/acs.bioconjchem.4c00353)
Supplement: Supplementary file 1 — bc4c00353_si_001.pdf [file bc4c00353_si_001.pdf]

REACTIVITY PROFILING FOR HIGH YIELDING YNAMINE-TAGGED OLIGONUCLEOTIDE CLICK CHEMISTRY BIOCONJUGATIONS

Frederik Peschke,[1,2] Andrea Taladriz-Sender,[1,2] Allan J.B. Watson,[3*] Glenn A. Burley[1,2*]

[1] Department of Pure and Applied Chemistry, University of Strathclyde. Thomas Graham Building, 295 Cathedral Street, Glasgow, G1 1XL, U.K. Email: glenn.burley@strath.ac.uk

[2] Strathclyde Centre for Molecular Bioscience, University of Strathclyde.

[3] EaStCHEM, School of Chemistry, University of Saint Andrews, North Haugh, St Andrews, Fife, United Kingdom, KY16 9ST. Email: aw260@st-andrews.ac.uk

Contents

REACTIVITY PROFILING FOR HIGH YIELDING YNAMINE-TAGGED OLIGONUCLEOTIDE CLICK CHEMISTRY BIOCONJUGATIONS 1

Contents 1

General Experimental Techniques and Procedures 2

1.1 Small Molecule Synthesis 3

1.2 Synthesized Oligonucleotides 4

HPLC Assay Protocol 8

1.3 Procedure for Oligonucleotide labelling and characterisation. 8

Synthesis and Analysis of Oligo-Protein Conjugates 10

1.4 Synthesis of BSA-Az 10

1.5 Oligo-Protein Conjugation 10

1.6 Oligo-Protein Purification and Analysis 11

1.7 Characterisation Data 13

Additional Figures 14

References 17

5. References 14

General Experimental Techniques and Procedures

All reagents and solvents were purchased from commercial sources and used without further purification. Thin layer chromatography (TLC) was carried out using Merck silica plates coated with fluorescent indicator UV254. TLC plates were analysed under 254 nm UV light or developed using potassium permanganate solution. Data analysis was performed in Microsoft Excel and data plotted with OriginPro®.

1X DPBS buffer was purchased from Sigma-Aldrich (SKU: D8662-100ML). Sulfo-Cy3-azide was purchased from Jena Bioscience (CLK-AZ119-5). Calfluor 488 was purchased from CRB Discovery (crb7008193j). Biotin-picolyl-azide was purchased from Sigma Aldrich (900912-50MG). BSA was purchased from Sigma-Aldrich (A7030-10G).

Column Chromatography (flash chromatography) was carried out on a puriFlash® X520S and Silia*Sep*™ columns. The exact conditions are reported in the experimental information for each compound.

NMR spectra (^{1}H and ^{13}C NMR) were obtained using Bruker spectrometers (400, 500 or 600 MHz). All chemical shifts (δ) were referenced at 7.26 (^{1}H) and 77.06 ppm (^{13}C) in $CDCl_3$. Coupling constants are reported in hertz (Hz) and multiplicities are abbreviated as follows: s = singlet, d = doublet, t = triplet, q = quartet and m = multiplet.

ICP-MS samples were analysed at the University of Leeds, School of Earth and Environment at the Inductively Coupled Plasma Analysis facility. The samples were analysed in a Thermo Scientific iCAPQc ICP-MS.

MALDI-ToF mass spectra were recorded using a Shimadzu Axima Confidence spectrometer, using 3-hydroxypicolinic acid (HPA) and ammonium citrate as the matrix. Spectra were recorded in positive mode (linear or reflectron).

1.1 Small Molecule Synthesis

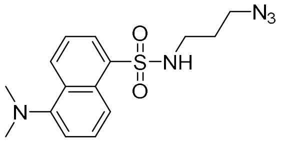

Dansyl-N3 (S1)

Dansyl chloride (121 mg, 449 µmol, 1 equiv) was dissolved in DCM (2 mL) and triethylamine (125 µL, 899 µmol, 2 equiv) was added. Then the reaction was cooled to 0 °C and 3-azido-1-propanamine (44.1 µL, 449 µmol, 1 equiv) was added and the reaction stirred in an ice bath for 2 h. The reaction mixture was directly injected onto a column (12 g silica) and purified by flash column chromatography (2 – 40% ethyl acetate in hexane over 10 CV). The fractions corresponding to the product where pooled, and the solvents evaporated under vacuum to obtain the desired product bright green oil (126 mg, 84%).

^{1}H NMR (500 MHz, $CDCl_3$) δ 8.56 (app dt, *J* = 8.5, 1.1 Hz, 1H), 8.28 – 8.24 (m, 2H), 7.58 (dd, *J* = 8.6, 7.6 Hz, 1H), 7.54 (dd, *J* = 8.6, 7.3 Hz, 1H), 7.20 (dd, *J* = 7.6, 0.9 Hz, 1H), 4.82 (t, *J* = 6.3 Hz, 1H), 3.26 (t, *J* = 6.4 Hz, 2H), 2.98 (q, *J* = 6.5 Hz, 2H), 2.90 (s, 6H), 1.66 (quint, *J* = 6.5 Hz, 2H).

^{13}C NMR (126 MHz, $CDCl_3$) δ 152.3, 134.5, 130.8, 130.1, 130.0, 129.7, 128.7, 123.4, 118.6, 115.4, 49.0, 45.6, 41.0, 29.0.

NMR spectra in agreement with literature values.[1]

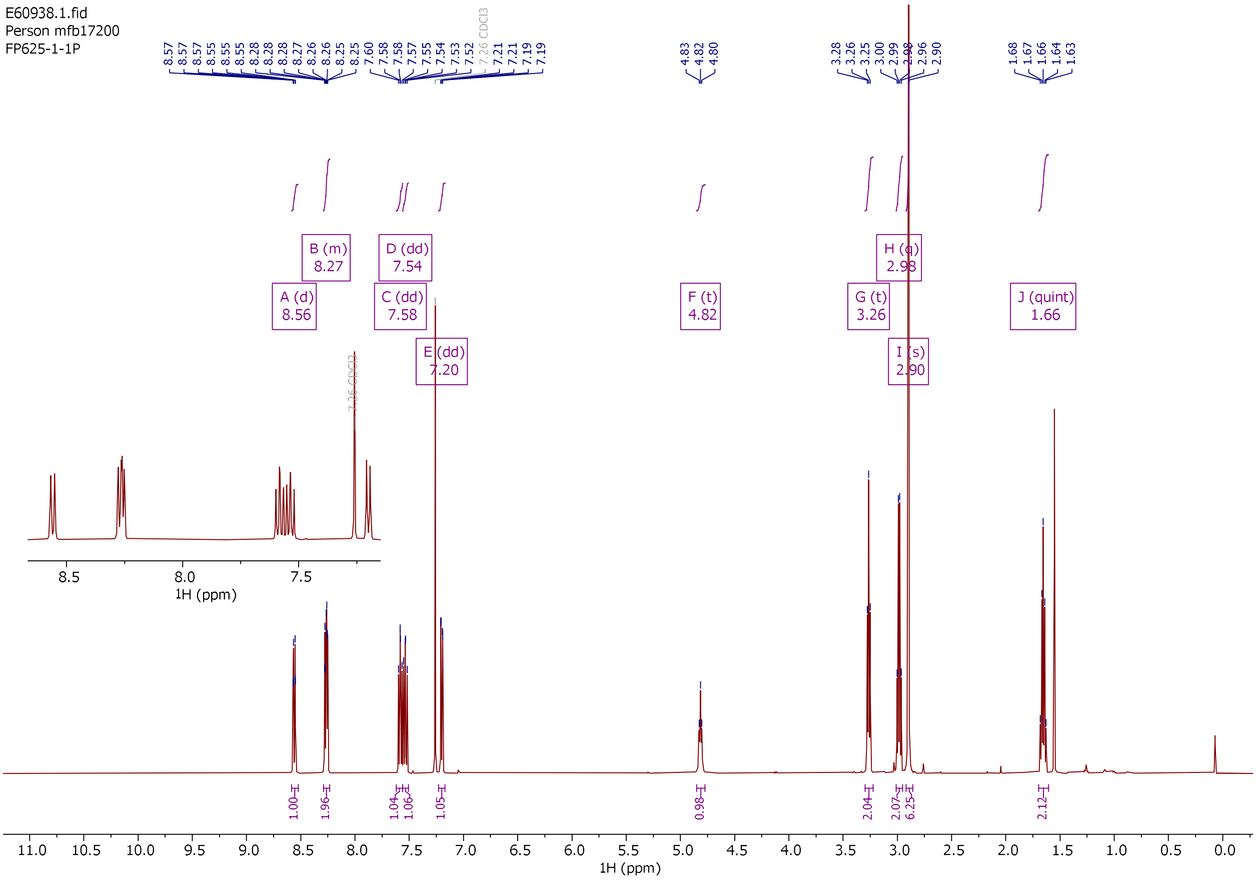

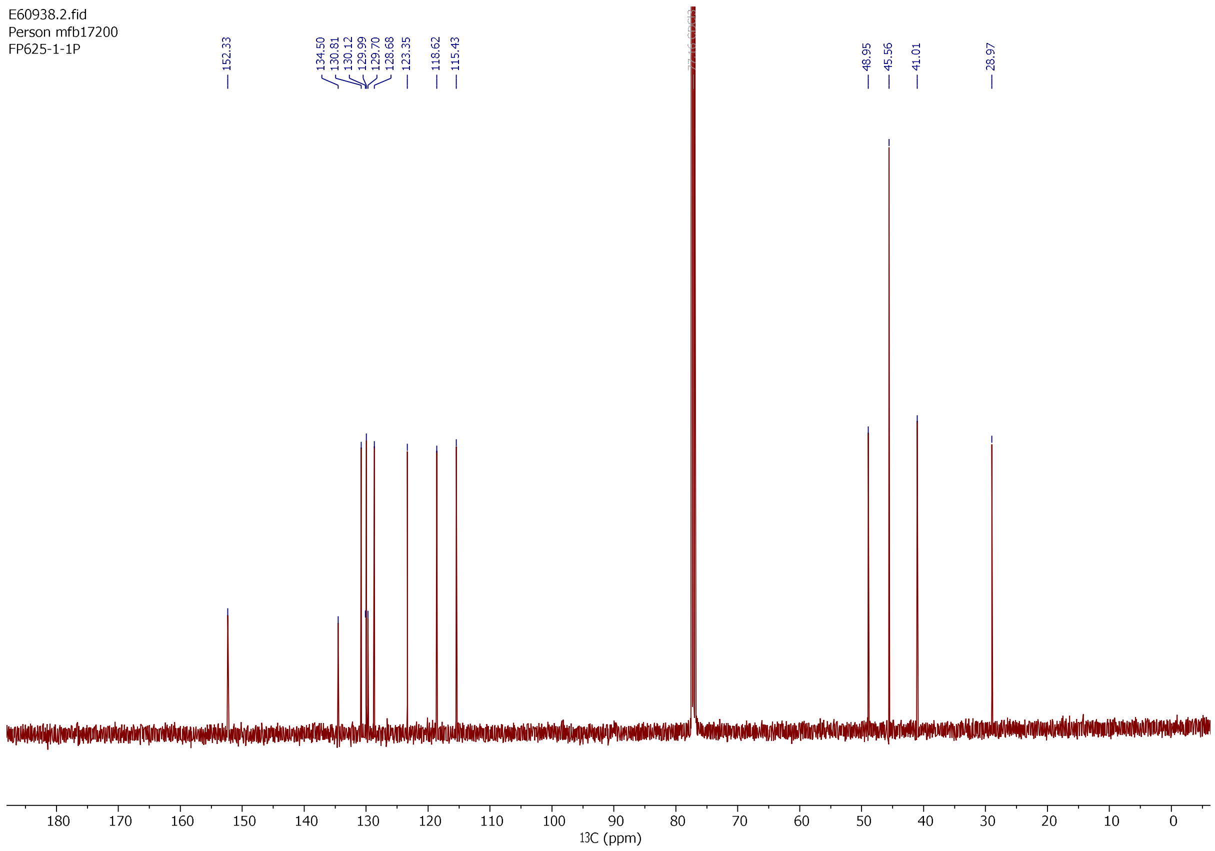

1.2 Synthesized Oligonucleotides

Phosphoramidites Structures:

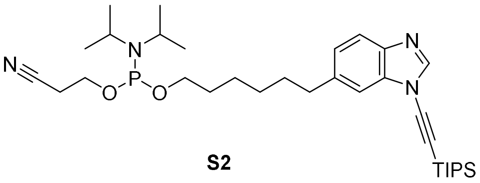

S2

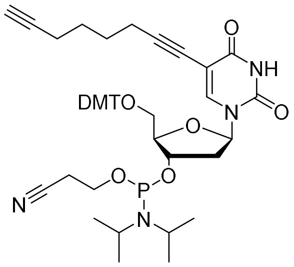

S3

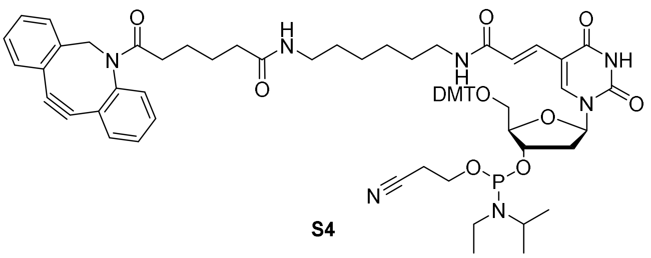

S4

Figure S1. Special phosphoramidites used in this study.

Oligonucleotide Structures:

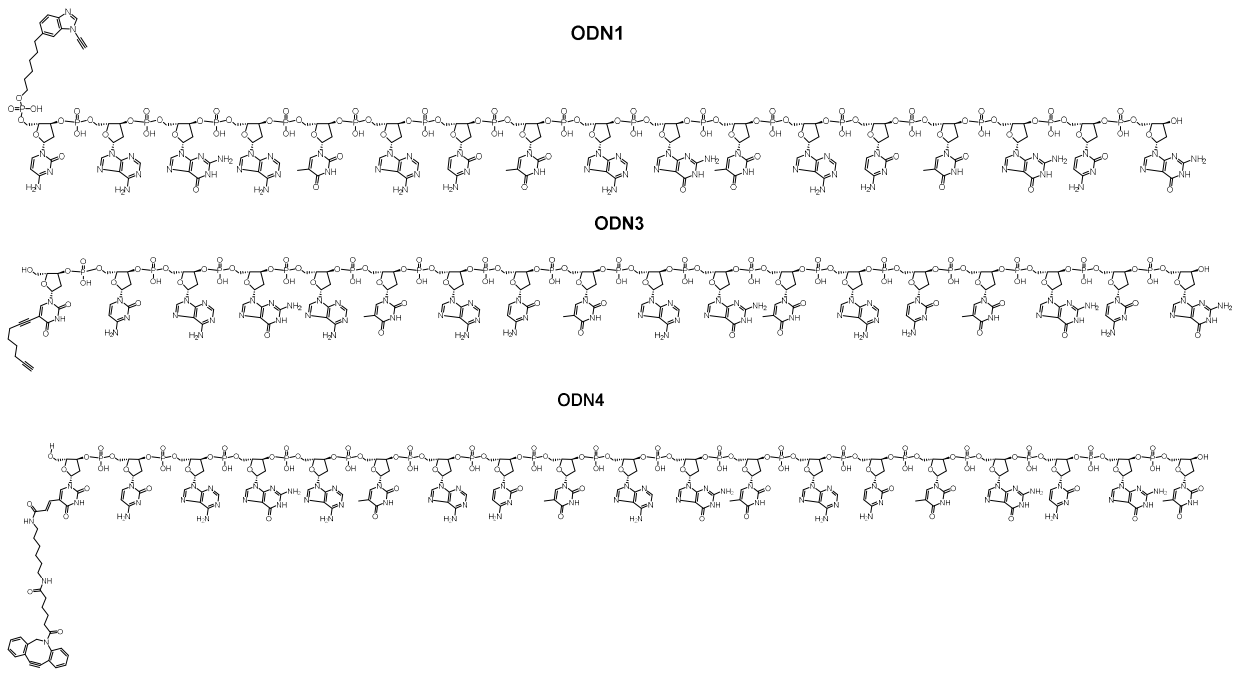

Figure S2. Structures of oligos used in this study.

Synthesis Protocol

ODN1, **ODN3** and **ODN4** were synthesized using standard solid phase oligonucleotide synthesis protocols on an ABI 392 synthesizer. Standard phosphoramidites and CPG supports loaded with standard nucleosides were purchased from LINK Technologies Ltd (Bellshill, UK). Phosporamidite **S2** was synthesised according to the procedure published in the literature.[2] Phosphoramidite **S3** and **S4** were purchased from Glen Research (C8-Alkyne-dT-CE Phosphoramidite, https://www.glenresearch.com/click-chemistry/10-1540.html and DBCO-dT-CE Phosphoramidite, https://www.glenresearch.com/click-chemistry/10-1539.html). For the modified phosphoramidite **S2, S3** and **S4** a longer coupling time of 5 min, 3 min and 12 min were used respectively.

After the solid phase synthesis was finished the TIPS protecting group in **ODN1** was removed on solid support. TBAF (50 µL, 1 M in THF) was mixed with 1.95 mL of acetonitrile and this mixture was taken up in a plastic syringe. The mixture was repeatedly passed over the column containing the CPG support for 2 min. The CPG support was washed with MeCN (3 × 5 mL) and dried with air.

Ammonia (DNA grade, 1.5 ml/mmol) was added, and the suspension was shaken for 16 h at room temperature. (ATTENTION: Higher Temperatures led to degradation of the ynamine modifiers and need to be avoided!). The supernatant was removed, and the CPG support was then washed with water (2 × 1.5 mL). The combined aqueous phase was concentrated under reduced pressure to obtain crude **ODN1, ODN3** and **ODN4** as a white solid. Oligonucleotides were purified by reverse-phase HPLC on a Dionex UltiMate 3000 System using a

Phenomenex Clarity Oligo-RP column (250 × 10 mm, 5 µm, 5 mL/min, A = 0.1 M TEAA in water (pH 7), B = 0.1 M TEAA, 80% MeCN in water (pH 7)), lyophilised and then desalted using a NAP-25 Sephadex column.

Purification Method

Dionex UltiMate 3000 Semi-prep system

Column Specifications: Phenomenex Clarity™ Oligo-RP, 250 × 10 mm, 5 µm

Column Temperature: 25 °C

Mobile Phase A: 0.1 M TEAA in water (pH 7)

Mobile Phase B: 0.1 M TEAA, 80% MeCN in water (pH 7)

Flow rate: 5 mL/min

Active Gradients (followed by wash at 95% B and re-equilibration in starting % B):

ODN1: 20 – 35% B in 12 mins

ODN3: 10 – 50% B in 13 mins

ODN4: 20 – 50% B in 12 mins

Purity Analysis Method

Thermo Scientific Vanquish Flex System

Column Specifications: Phenomenex bioZen™ Oligo column, 50 × 4.6 mm, 2.6 µm.

Column Temperature: 50 °C

Mobile Phase A: 0.1 M TEAA in water (pH 7)

Mobile Phase B: 0.1 M TEAA, 80% MeCN in water (pH 7)

Flow rate: 1.2 mL/min

Injection Volume: 10 µL

Gradient:

**Time** (min)	**B%**
0 – 6.8	5 – 50
6.8 – 7	50 – 95
7 – 8	95
8 – 8.2	95 – 5
8.2 – 9.5	5

Oligonucleotide Sequence and characterisation

Table S1 Synthesised Oligonucleotides

Oligo	Modification	Sequence 5'-3'	Calcd Mass (M+H)	Found (MALDI)	Purity (HPLC)
**ODN1**	Ynamine (Yn)	Yn-CAGATACTAGTACTGCG	5497.02	5497.99	96%
**ODN3**	Alkyne (Alk)	Alk-CAGATACTAGTACTGCG	5587.01	5586.73	99%
**ODN4***	DBCO	DBCO-CAGATACTAGTACTGCGT	6270.25	6270.04	94%

Note: We observed **ODN4 degradation after freeze drying following the purification and under prolonged storage (>1 month) as an aqueous solution in the freezer at -20 °C.*

Oligonucleotide conjugates synthesised and azides used for labelling.

Azides

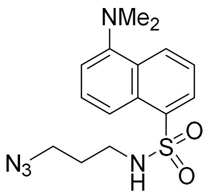

S1

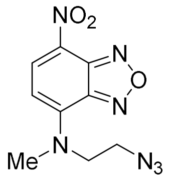

S5

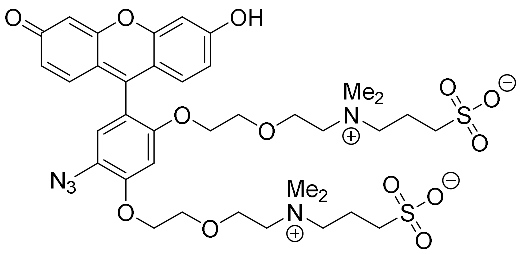

S6

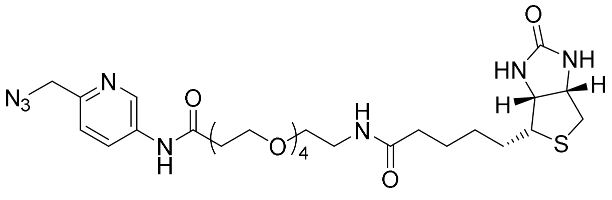

S7

Figure S3. Azides used in the conjugation reactions in Figure 5.

Table S2. ODN and azides reactants and the corresponding oligonucleotide conjugate products

Oligo	5' Modification	Azide	Oligo conjugate product
**ODN1**	Ynamine (Yn)	S5	ODN5
		S1	ODN6
		S6	ODN7
		S7	ODN8
**ODN3**	Alkyne (Alk)	S5	ODN9
		S1	ODN10
		S6	ODN11
		S7	ODN12
**ODN4**	DBCO	S5	ODN13
		S1	ODN14
		S6	ODN15
		S7	ODN16

MALDI Characterisation of products obtained in Figure 5

For the preparation of MALDI samples the ODN solution was desalted using a Ziptip® according to the manufacturer's instructions (Technical Note 225: Sample Preparation of Oligonucleotides Prior to MALDI-TOF MS using ZipTipC18 and ZipTipµ-C18 Pipette Tips) and eluted with 5 µL (H_2O/MeCN = 1:1) and subsequently analysed by MALDI-TOF (Shimadzu Axima Performance™; Linear mode; matrix: 3-HPA (50 mg/mL), ammonium citrate dibasic (50 mg/ml) in H_2O/MeCN = 1:1).

Table S3. MALDI-ToF masses of oligos synthesised in Figure 5.

ODN	Calc Mass (M+H)	Found (MALDI-ToF)
ODN5	5760.09	NDB degradation in MALDI
ODN6	5830.14	5830.09
ODN7	6332.29	6331.75
ODN8	6119.31	6119.67
ODN13	6533.32	NDB degradation in MALDI
ODN14	6603.32	No signal obtained
ODN15	7105.52	7105.50
ODN16	6892.54	6893.70

*Note: Due to poor conversion no masses could be obtained for the alkyne oligo (**ODN3**) products (**ODN9-12**).*

HPLC Assay Protocol

1.3 Procedure for Oligonucleotide labelling and characterisation.

Stock solutions of $Cu(OAc)_2$, NaAsc and GSH were freshly prepared the experiment day. Stock solutions of alkynes, azides and THTPA were stored in the fridge and used up to 5 days. After purification and desalting the oligonucleotides were dissolved in ultrapure H_2O (MiliQ) and the concentration measured using a Thermo Scientific Nanodrop 1000. Extinction

coefficients were calculated as follows: 173880 M^{-1} cm^{-1} (**ODN1**), 177100 M^{-1} cm^{-1} (**ODN3**) and 195600 M^{-1} cm^{-1} (**ODN4**); calculated using IDT OligoAnalyzer™ Tool plus 18800 for **ODN4** (*Source:* Glen Research).

Exemplary stock solutions:

[Sulfo-Cy3-azide **2**] = 2 mM in H_2O

[GSH] = 5 mM in H_2O

[$Cu(OAc)_2$] = 0.5 mM in H_2O

[NaAsc] = 20 mM in H_2O

[THPTA] = 5 mM in H_2O

General Procedure:

To and HPLC vial (Waters™ QuanRecovery© with MaxPeak HPS, SKU: 186009186; *Note: inconsistent peak areas were obtained when using normal polypropylene vials*) was added organic solvent (i.e. MeOH) and aqueous buffer. The vial was then placed in the autosampler (25 °C). Azide, $Cu(OAc)_2$, THPTA, NaAsc or GSH were added via UPD liquid handling protocol and the reaction was sampled every 30 mins for the specified amount of time (see example below).

Example (10% MeOH in H_2O (20mM $MgCl_2$). Final volume = 100 µL):

Substrate	Desired Concentration (µM)	µL added from Stock Solution
Ynamine-Oligo **ODN1**	10	4.3
Sulfo-Cy3-azide **2**	20	4
MeOH	N/A	10
H_2O (20 mM $MgCl_2$)	N/A	72.7
THPTA	200	4
$Cu(OAc)_2$	20	4
NaAsc	200	1

Instrument Specifications:

Thermofisher Vanquish Flex System

Column Specifications: Phenomenex Biozen® C18, 50 x 2.1 mm, 2.6 µm

Column Temperature: 60 °C

Mobile Phase A: 50 mM HFIP, 5 mM DIPEA in H_2O

Mobile Phase B: 50 mM HFIP, 5 mM DIPEA in H_2O/MeCN (1:1)

Flow rate: 0.6 mL/min

Injection volume: 3 µL

Gradient Profile:

**Time** (min)	**B%**
0 – 6	10 – 35
6 – 6.2	35 – 100
6.2 – 7.0	100
7.0 – 7.5	100 – 10
7.5 – 9.5	10

UPD Liquid Handling Protocol:

Most reactions were set up using the build in UDP-handling protocol of the Thermo Scientific™ Vanquish Flex autosampler according to the protocol below. This afforded a high level of consistency.

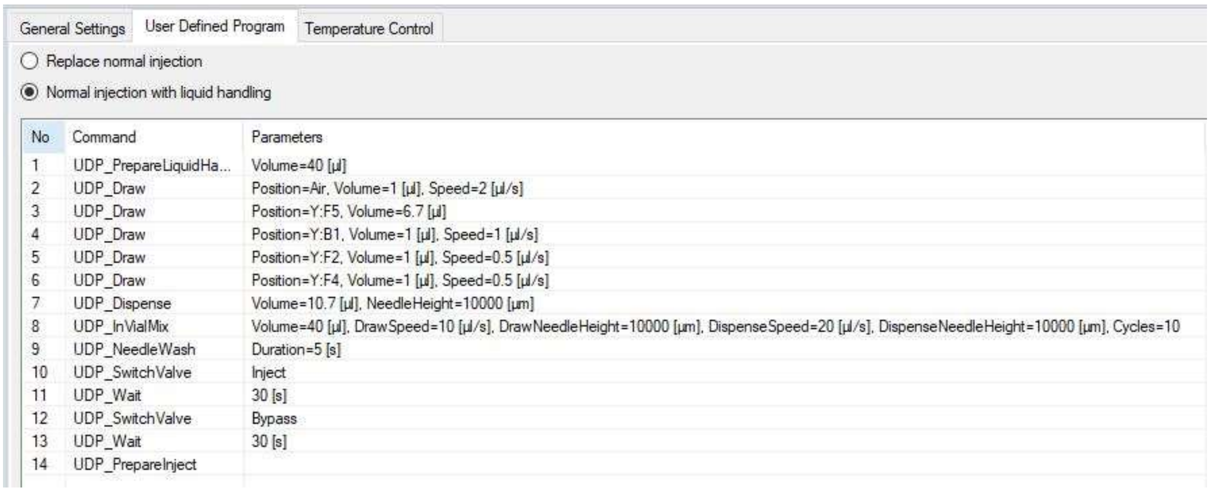
General Settings | User Defined Program | Temperature Control

○ Replace normal injection

◉ Normal injection with liquid handling

No	Command	Parameters
1	UDP_PrepareLiquidHa...	Volume=40 [µl]
2	UDP_Draw	Position=Air, Volume=1 [µl], Speed=2 [µl/s]
3	UDP_Draw	Position=Y:F5, Volume=6.7 [µl]
4	UDP_Draw	Position=Y:B1, Volume=1 [µl], Speed=1 [µl/s]
5	UDP_Draw	Position=Y:F2, Volume=1 [µl], Speed=0.5 [µl/s]
6	UDP_Draw	Position=Y:F4, Volume=1 [µl], Speed=0.5 [µl/s]
7	UDP_Dispense	Volume=10.7 [µl], NeedleHeight=10000 [µm]
8	UDP_InVialMix	Volume=40 [µl], DrawSpeed=10 [µl/s], DrawNeedleHeight=10000 [µm], DispenseSpeed=20 [µl/s], DispenseNeedleHeight=10000 [µm], Cycles=10
9	UDP_NeedleWash	Duration=5 [s]
10	UDP_SwitchValve	Inject
11	UDP_Wait	30 [s]
12	UDP_SwitchValve	Bypass
13	UDP_Wait	30 [s]
14	UDP_PrepareInject	

Synthesis and Analysis of Oligo-Protein Conjugates

1.4 Synthesis of BSA-Az

BSA-Az was synthesised by incubating BSA (10 mg, Sigma-Aldrich, A7030-10G) with maleimide-PEG3-azide (1.1 equiv, 3 µL, ~75 mM in DMF, Thermo Scientific Chemicals, J65984.MC) for 3 h in 1.5 mL 1X DPBS. The excess small molecule was removed via spin filtration on a Vivaspin® 500 (10k MWCO) and the conjugate analysed by LC-HRMS (see for method below). The concentration was determined on the ThermoFisher Nanodrop 1000 using the pre-determined program.

1.5 Oligo-Protein Conjugation

Exemplary stock solutions:

[**BSA-Az**] = 480 µM in H_2O

[**ODN1**] = 150 µM in H_2O

[GSH] = 5 mM in H_2O

[$Cu(OAc)_2$] = 5 mM in H_2O

General Procedure:

To and HPLC vial (Waters™ QuanRecovery© with MaxPeak HPS, SKU: 186009186; *Note: inconsistent peak areas were obtained when using normal polypropylene vials*) was added GSH and $Cu(OAc)_2$ (pipetted into one drop) followed by organic solvent (pipetted in a separate drop). To the rim of the vial was then added **BSA-Az** and **ODN1** in separate drops and all four drops were then combined by pipetting and mixing 50 mM HEPES (pH 8) into the vial.

Example (5% DMSO in HEPES buffer; final volume = 50 µL):

**Substrate**	**Desired Concentration (µM)**	**µL added from Stock Solution**
ODN1	10	3
BSA-Az	40	4.2
DMSO	N/A	2.5
50 mM HEPES (pH 8)	N/A	34.3
$Cu(OAc)_2$	20	5
GSH	200	1

Anion Exchange Chromatography Analysis:

Thermofisher Vanquish Flex System

Column Specifications: Waters Gen-Pak™ FAX 100 × 4.6 mm, 2.5 µm

Column Temperature: 30 °C

Mobile Phase A: H_2O

Mobile Phase B: 1.25M NaCl

Mobile Phase C: 200 mM TRIS in H_2O (pH 8)

Mobile Phase D: Acetonitrile

Flow rate: 0.75 mL/min

Injection volume: 5 µL

Gradient Profile:

**Time** (min)	**B%**	**C%**	**D%**
0 – 5	10 – 70	10	10
5 – 6.8	70	10	10
6.8 – 7.0	70 – 10	10	10
7.0 – 9.5	10	10	10

1.6 Oligo-Protein Purification and Analysis

Purification by AEX:

Thermofisher Vanquish Flex System

Column Specifications: Thermo Scientific Proswift™ WAX-1S 50 × 4.6 mm, monolith

Column Temperature: 30 °C

Mobile Phase A: H_2O

Mobile Phase B: 1.25M NaCl

Mobile Phase C: 200 mM TRIS in H_2O (pH 8)

Mobile Phase D: Acetonitrile

Flow rate: 1 mL/min

Injection volume: 25 µL

Gradient Profile:

**Time** (min)	**B%**	**C%**	**D%**
0 – 5	10 – 70	10	10
5 – 6.8	70	10	10
6.8 – 7.0	70 – 10	10	10
7.0 – 9.5	10	10	10

Analysis by HRMS:

LC-MS grade water (Optima™; 10728098) was purchased from Fisher Scientific. LC-MS grade acetonitrile (Supelco LiChrosolv®, 1000291000) and LC-MS grade ammonium acetate (Supelco LiChropur™, 73594-25G-F) were purchased from Sigma-Aldrich. Purified conjugates were buffer exchanged (3×) into 50 mM ammonium acetate using Vivaspin® 500 (10k MWCO) concentrators. HRMS was performed on a Thermo Exactive Plus Orbitrap coupled to a Vanquish Flex UHPLC. Denaturing SEC-MS analysis was carried out using a Thermo Scientific™ NativePAC OBE-1 SEC column using a flow rate of 50 µL/min. The mobile phase was 50 mM ammonium acetate in 25% acetonitrile/water. Spectra were deconvoluted using UniDec.[3] UniDec parameters: Charge Range 1 – 100, Mass Range 60000 – 80000 Da, Sample Mass Every (Da): 1, Peak FWHM (Th): 0.3.

1.7 Characterisation Data

BSA-Az (expected: 66798, found 66798):

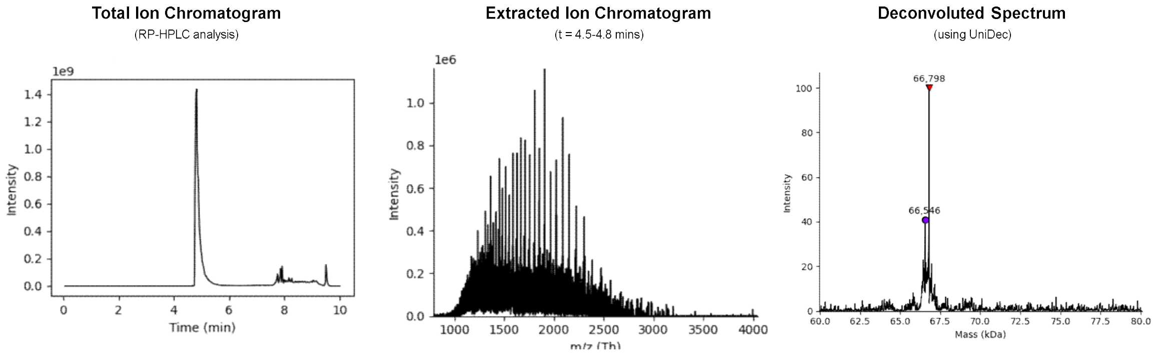

Figure S4. Characterisation of azide modified BSA (BSA-Az). BSA-Az was analysed by RPLC-MS instead of SEC-MS. Column: Phenomenex BioZen Widepore C4, 50 x 2.1 mm, 2.6 um. Gradient 5% B for 2min, then 5 - 95% B in 5min, followed by a wash (95% B) and equilibration (5% B). A = 0.1% formic in H_2O. B = 0.1% formic in MeCN. Deconvolution was performed using UniDec (see "*Analysis by HRMS*" above).

Ynamine-CuAAC oligo-protein conjugate (expected: 72297, found 72357 M+AcOH):

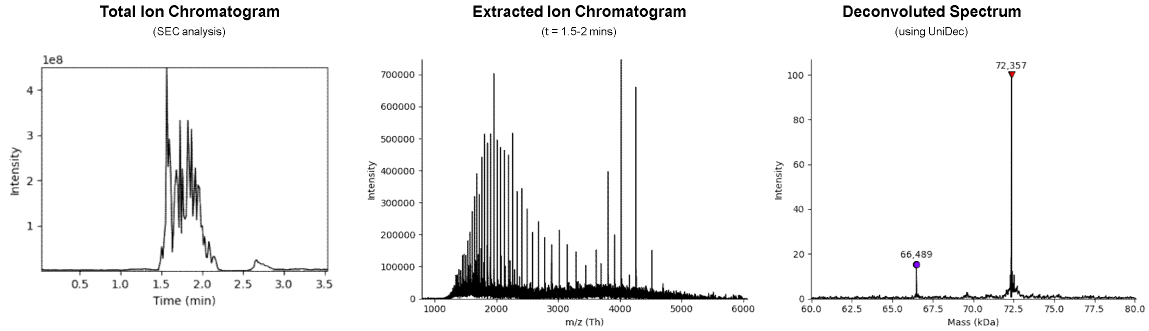

Figure S5. Characterisation of the Ynamine-oligo BSA conjugate. For conditions see "*Analysis by HRMS*" above.

DBCO-SPAAC oligo-protein conjugate (expected: 73069, found 72369):

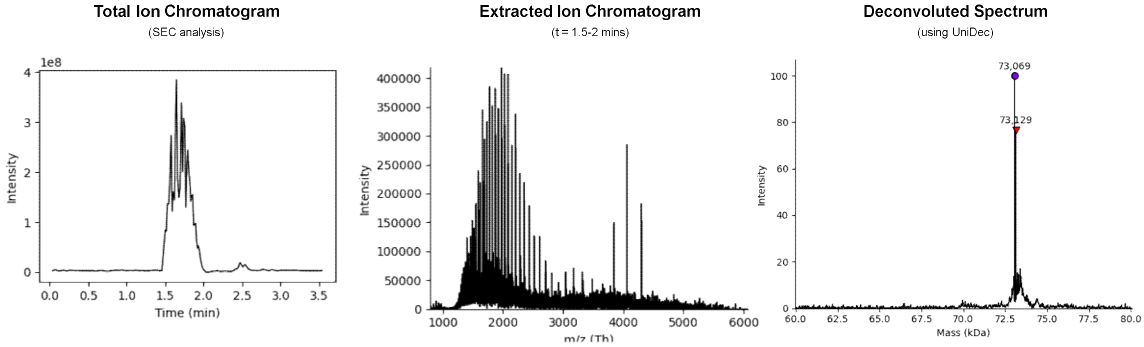

Figure S6. Characterisation of the DBCO-oligo BSA conjugate. For conditions see "*Analysis by HRMS*" above.

Note: Not enough material was obtained to characterize the alkyne-CuAAC oligo-protein conjugate.

Additional Figures

Effect of co-solvent and buffer on oligonucleotide reactivity

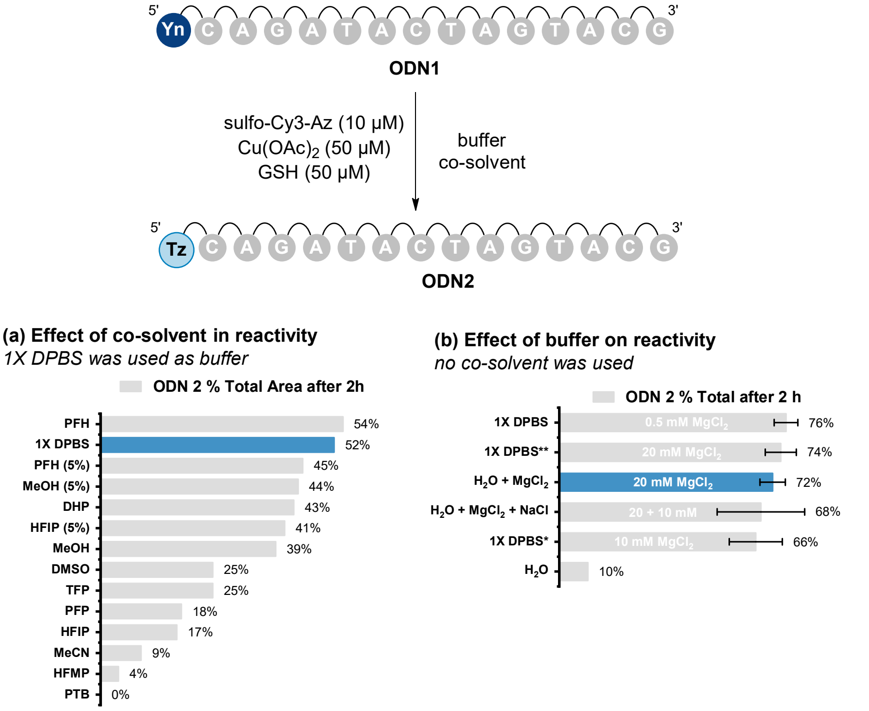

Figure S7. Ynamine-CuAAC reactivity using **ODN1** and sulfo-Cy3-azide **2** is influenced by buffer. No co-solvent and using H_2O (20 mM $MgCl_2$) were chosen due to simplicity.

Stability of Alkyne ODN3 in the presence of Cu/THPTA/NaAsc

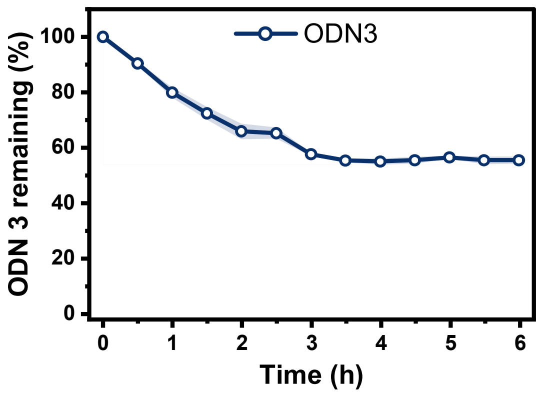

Figure S8. Stability of **ODN3** in the presence of Cu/THPTA/NaAsc. Similar degradation to **ODN1** is observed. *Conditions:* **ODN3** (10 µM), azide **S7** (20 µM), $Cu(OAc)_2$ (20 µM), THPTA (100 µM), NaAsc (200 µM), 10% MeOH in H_2O (20 mM $MgCl_2$).

Design of Experiment

Table S4. Concentrations of THPTA and NaAsc tested during the DoE. *Conditions:* **ODN1** (10 μM), $Cu(OAc)_2 \cdot H_2O$ (10 μM), Sulfo-Cy3-azide (20 μM), THPTA and NaAsc as specified in H_2O (20 mM $MgCl_2$), rt, 2.5h.

Entry	[THPTA] (μM)	[NaAsc] (μM)
1	10	100
2	10	1000
3	50	100
4	50	1000
5	30	500
6	30	500

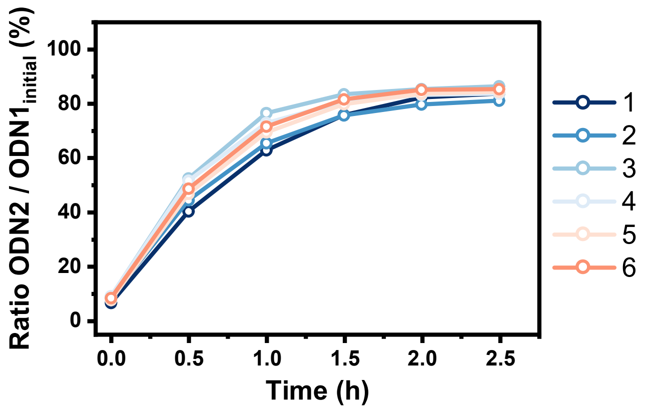

Figure S9. Time course of the DoE reactions between **ODN1** and azide **2**.

Conventional Alkyne-Oligo CuAAC

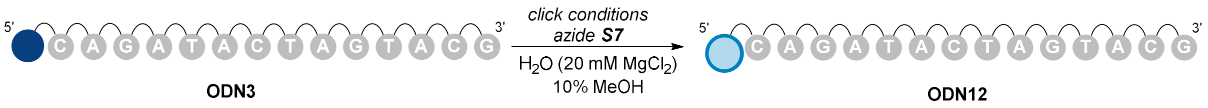

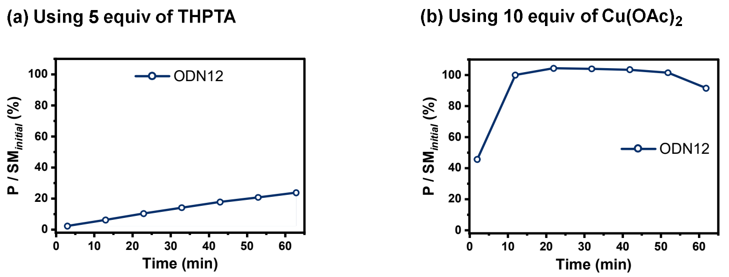

Figure S10. (a) Reaction of **ODN3** with picolyl azide (**S7**) using 5 equiv of THPTA instead of 10 equiv THPTA to test for ligand inhibition. The reaction is slightly faster with 5 equiv of THPTA. *Conditions:* **ODN3** (10 μM), azide **S7** (20 μM), $Cu(OAc)_2$ (20 μM), THPTA (100 μM), NaAsc (200 μM), 10% MeOH in H_2O (20 mM $MgCl_2$). (b) Reaction of **ODN3** with picolyl azide (**S7**) using 20 equiv of $Cu(OAc)_2$ to push the reaction to completion. Reactivity now on par with the ynamine. *Conditions:* **ODN3** (10 μM), azide **S7** (20 μM), $Cu(OAc)_2$ (200 μM), THPTA (1 mM), NaAsc (2 mM), 10% MeOH in H_2O (20 mM $MgCl_2$).

Optimisation of Oligo-Protein Reaction

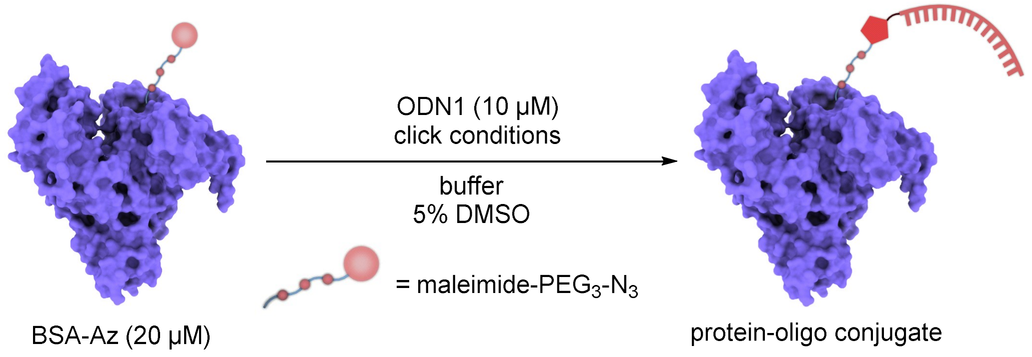

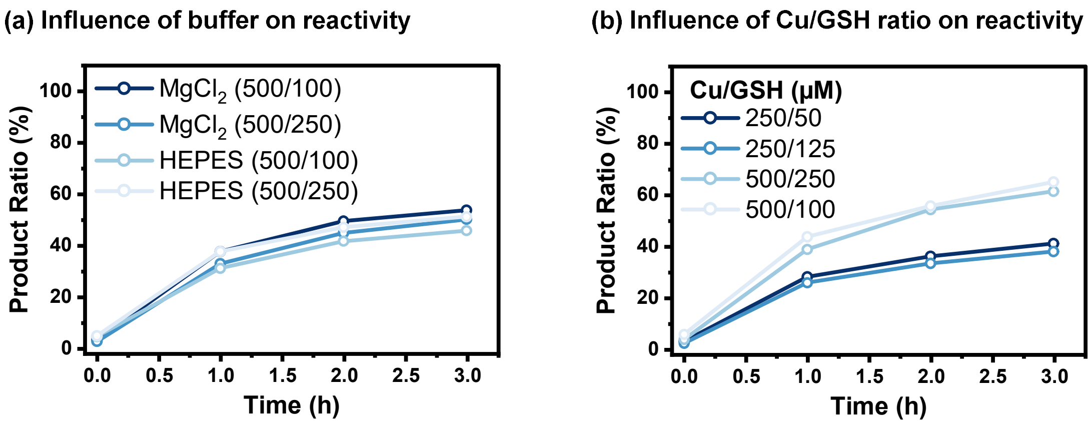

Figure S11. Optimisation of buffer and Cu/GSH ratio for the oligo-protein conjugation. (a) Reactivity is similar between H_2O (20 mM $MgCl_2$) and 50 mM HEPES (pH 8). (b) Cu/GSH has little influence on reactivity. Doubling [Cu] leads to a larger increase in reactivity. To ensure protein solubility and reactivity 50 mM HEPES (pH 8) and [$Cu(OAc)_2$]/[GSH] = 500/100 μM were chosen as the final conditions.

DBCO-SPAAC Oligo-Protein Conjugate

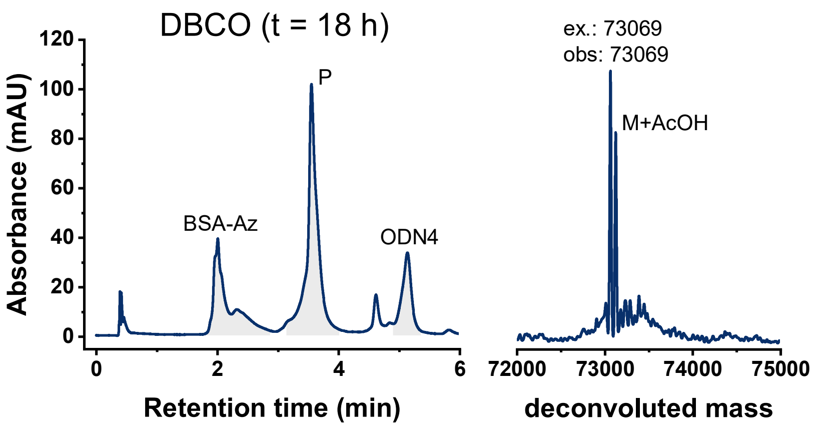

Figure S12. Analytical AEX trace after 18 h and HRMS of the AEX purified oligo-protein conjugate. The M+AcOH adduct was also observed as was the case the ynamine oligo-protein conjugate.

References

(1) Huang, C.-J.; Chang, F.-C. Using Click Chemistry To Fabricate Ultrathin Thermoresponsive Microcapsules through Direct Covalent Layer-by-Layer Assembly. *Macromolecules* **2009**, *42* (14), 5155-5166. DOI: 10.1021/ma900478n.

(2) Peschke, F.; Taladriz-Sender, A.; Andrews, M. J.; Watson, A. J. B.; Burley, G. A. Glutathione Mediates Control of Dual Differential Bio-orthogonal Labelling of Biomolecules. *Angew. Chem. Int. Ed.* **2023**, *62* (50), e202313063. DOI: https://doi.org/10.1002/anie.202313063.

(3) Marty, M. T.; Baldwin, A. J.; Marklund, E. G.; Hochberg, G. K. A.; Benesch, J. L. P.; Robinson, C. V. Bayesian Deconvolution of Mass and Ion Mobility Spectra: From Binary Interactions to Polydisperse Ensembles. *Anal. Chem.* **2015**, *87* (8), 4370-4376. DOI: 10.1021/acs.analchem.5b00140.
